# Systematic Analysis and Functional Validation of Citrus Pectin Acetylesterases (CsPAEs) Reveals that CsPAE2 Negatively Regulates Citrus Bacterial Canker Development

**DOI:** 10.3390/ijms21249429

**Published:** 2020-12-11

**Authors:** Qiang Li, Jia Fu, Xiujuan Qin, Wen Yang, Jingjing Qi, Zhengguo Li, Shanchun Chen, Yongrui He

**Affiliations:** 1 Citrus Research Institute, Southwest University/Chinese Academy of Agricultural Sciences, Chongqing 400712, China; fjduolaimi@126.com (J.F.); qinxiujuan-cric@foxmail.com (X.Q.); yangwen114429@foxmail.com (W.Y.); qijingjing-cric@foxmail.com (J.Q.); citgvd@cric.cn (S.C.); 2 Key Laboratory of Plant Hormones and Development Regulation of Chongqing, School of Life Sciences, Chongqing University, Chongqing 401331, China; zhengguoli@cqu.edu.cn

**Keywords:** pectin acetylesterase (PAE), *Xanthomonas citri* subsp. *citri* (*Xcc*), citrus bacterial canker (CBC), *Citrus sinensis*

## Abstract

The present study was designed to serve as a comprehensive analysis of *Citrus sinensis* (*C. sinensis*) pectin acetylesterases (CsPAEs), and to assess the roles of these PAEs involved in the development of citrus bacterial canker (CBC) caused by *Xanthomonas citri* subsp. *citri* (*Xcc*) infection. A total of six *CsPAEs* were identified in the genome of *C. sinensis*, with these genes being unevenly distributed across chromosomes 3, 6, and 9, and the unassembled scaffolds. A subset of *CsPAEs* were found to be involved in responses to *Xcc* infection. In particular, *CsPAE2* was identified to be associated with such infections, as it was upregulated in CBC-susceptible variety Wanjincheng and inversely in CBC-resistant variety Calamondin. Transgenic citrus plants overexpressing *CsPAE2* were found to be more susceptible to CBC, whereas the silencing of this gene was sufficient to confer CBC resistance. Together, these findings provide evolutionary insights into and functional information about the CsPAE family. This study also suggests that *CsPAE2* is a potential candidate gene that negatively contributes to bacterial canker disease and can be used to breed CBC-resistant citrus plants.

## 1. Introduction

All living beings must be able to efficiently and effectively detect and respond to danger [1]. The primary plant cell wall functions as a major protective barrier that can prevent pathogenic infection [2,3]. These cell walls are composed of heavily cross-linked polysaccharide polymer networks [2,4,5,6], with pectin, cellulose, and hemicellulose fibrils forming a matrix that serves as a barrier that can only be penetrated via mechanical force or the secretion of specific digestive enzymes [3]. In addition to its barrier function, the cell wall is essential for plant cells to detect and respond to biotic stress. Multiple different receptors and other sensory molecules are present within the plasma membranes of plant cells, allowing for the detection of apoplastic infections and the resultant induction of appropriate symplastic immune responses [7]. A number of different molecules are capable of binding wall-associated receptors in order to induce such immune responses, including cell wall-derived molecules, DNA fragments, and misfolded proteins [8]. Many of these elicitor compounds are derived from pectin, which is composed of a rhamnogalacturonan or homogalacturonan backbone and is the most prevalent polysaccharide within the cell wall of nongraminaceous plants [9].

Pectin composes up to a third of the cell wall by mas, and can be modified via C2 and/or C3 galacturonic acid residue acetylation [10]. The specific acetylation and methylation patterns present on pectin fragments ultimately determine the degree to which they function as elicitors of immune responses, and pectin acetylesterase (EC 3.1.1.6, PAE) can cleave pectin acetylester bonds to modulate these patterns [10,11,12,13]. Pectin de-esterification can result in acetate and/or methanol release, allowing these compounds to be readily incorporated back into metabolic pathways within the plant. This can also result in the accumulation of negatively charged carboxyl groups, potentially leading to a drop in pH that may impact the activity of various apoplastic proteins and ion channels, including wall-loosening expansins [14,15]. Pectin de-esterification also impacts the apoplastic reactive oxygen species’ (ROS) homeostasis, which has not been sufficiently studied [16]. Multiple independent reports have found that pectin configurations are key determinants of the ability of plants to muster effective immune responses against pathogens, and as such there is clear value in further studying related regulatory pathways in an effort to identify novel disease management strategies [17]. At present, however, only a limited number of studies have explored plant cell wall-mediated immune responses at the metabolic and transcriptional levels, and these studies have primarily focused on such responses in the context of pectin demethylation.

In the CAZy database, plant PAEs are members of the CE13 carbohydrate esterase family [18]. Recent improvements in plant genomic datasets have led to more widespread PAE annotation and study in different plant species. However, few studies have explored PAE physiological functions to date, or the evolution, function, and structure of the *PAE* gene family in plants [13,19,20]. These studies give the insight that a lower plant has fewer pectin-related gene family members compared to Arabidopsis, and only one ancestral *PAE* in the earliest land plant [11,20,21]. In addition, there have not been sufficient studies of the function and expression of *PAEs* in different plants, and the roles in plant development of these proteins remain uncertain. The overexpression of *PtPAE2* in tobacco has been shown to have a significant adverse impact on floral development, leading to decreased pollen formation and resultant sterility [12]. In addition, plants bearing *AtPAE8* and *AtPAE9* mutations have been found to exhibit short inflorescence stems [19].

Past studies demonstrated that PAEs likely play important regulatory roles in plant responses to biotic stressors. For example, *AtPAE2* and *AtPAE4* were upregulated in response to biotic stressors, suggesting they may be key regulators of plant defense responses [11]. In response to pathogenic infection and other stressors, the cell wall undergoes a number of morphological and physiological changes regulated by expansins (EXPs) [22], PAEs [11], and xyloglucan endotransglucosylase/hydrolases (XTHs) to product oligogalacturonides (OGAs), which are fragments of the homogalacturonan domains of pectin [2,23]. The OGAs released by the cell wall can function as signaling intermediates, modulating ROS homeostasis to activate plant immune responses [24,25,26,27]. These OGAs are damage-associated molecular patterns (DAMPs) [28], and their accumulation can induce microbial resistance in Arabidopsis and tobacco [29]. In wheat, infection level of *Blumeria graminis* could be induced by acetylated OGAs and non-acetylated OGAs, which provides evidence for elicitation and protection effects of preventive treatments with OGAs in wheat and for new properties of acetylated OGAs [24]. More research into the role of pectin acetyl esterification in plant immunity is, however, still needed [3].

Citrus bacterial canker (CBC) caused by *Xanthomonas citri* subsp. *citri* (*Xcc*) is a serious bacterial disease [30,31,32,33]. In the present study, we conducted the comprehensive in silico annotation of *C. sinensis PAEs* [34,35]. We further conducted a functional analysis of *PAE* genes and explored their relevance to CBC resistance. The functions involved in CBC development were then validated by reverse genetics strategies. Together, our findings highlight novel potential approaches to reducing the CBC susceptibility.

## 2. Results

### 2.1. Six CsPAEs Were Identified and Annotated in C. sinensis Genome

Through exhaustive data mining and annotation efforts, we were able to identify and characterize six *CsPAE* genes named *CsPAE1*–*6* (Table 1). Of these genes, all six were predicted based upon Citrus annotation project (CAP) database, and five were also predicted by Phytozome. In order to validate these putative *CsPAEs*, the best expressed sequence tag (EST) hits were extracted from the EST dataset (NCBI), confirming that all six of these *CsPAEs* were identified with a total of 21 ESTs (Table S1). Among the six, *CsPAE1* possesses the most ESTs (12). The *PAE* genes of Wanjincheng were cloned and sequenced according to the *PAEs* in the reference genome. We finally found that only *CsPAE4* contained a 2-base difference from the reference gene in CAP (CAP ID: Cs6g06280.1). The gene, CsPAE2 coding sequences (CDS), and protein sequences of CsPAEs are included in Table S2. The isoelectric point (PI), means of annotation, and molecular weight (MW) are compiled in Table 1. The *CsPAE* genes encodes 386 (*CsPAE2*) to 423 (*CsPAE6*) amino acid residues with MW 42542.05–49247.08 Dalton. CsPAE2 and CsPAE4 contain more acidic amino acids, making the proteins appear acidic (PI < 7), while more basic amino acids made CsPAE1, 3, 5, and 6 appear basic (PI > 7).

### 2.2. Phylogenetic Analysis of CsPAEs

**Figure 1:**
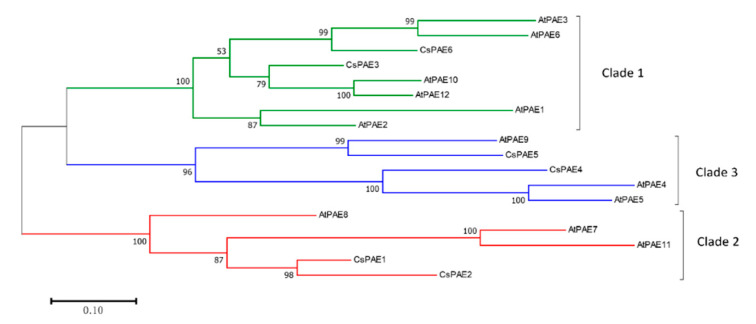
Maximum-Likelihood (ML) phylogeny of all the PAEs from *C. sinensis* and *A. thaliana*. An ML tree was constructed based on the PAE amino acid sequences of *A. thaliana* and *C. sinensis* (12 and 6, respectively) with MEGA V7.0 (bootstrap = 500, Poisson model). Branches are drawn to scale, with length corresponding to the number of substitutions per site. Sub-family assignments are shown on the right. Clades are color-coded as indicated.

In order to study the phylogenetic relationships of PAEs between organisms, phylogeny of CsPAEs were conducted based upon comparing their full amino acid sequences to those of AtPAEs. The resultant ML phylogenetic tree indicated that CsPAEs can be separated into three distinct clades (clades 1–3) in accordance with the clades used for AtPAE identification (Figure 1) [11]. Specifically, CsPAE3 and CsPAE6 were in clade 1, CsPAE4 and CsPAE5 were in clade 2, and CsPAE1 and CsPAE2 were in clade 3. Based on the phylogeny, genes in pairs CsPAE5–AtPAE9, CsPAE4–AtPAE4/5, and CsPAE3–AtPAE3/6 displayed close relationships that indicate the interspecific homologies. The phylogenetic tree also showed the intraspecific homology between citrus and Arabidopsis. Four pairs of homologous PAEs (AtPAE3 and AtPAE6, AtPAE10 and AtPAE12, AtPAE4 and AtPAE5, AtPAE7 and AtPAE11) were detected in Arabidopsis, whereas only one pair (CsPAE1 and CsPAE2) was in citrus.

### 2.3. Conserved Domains and Secondary Structures of CsPAEs

**Figure 2:**
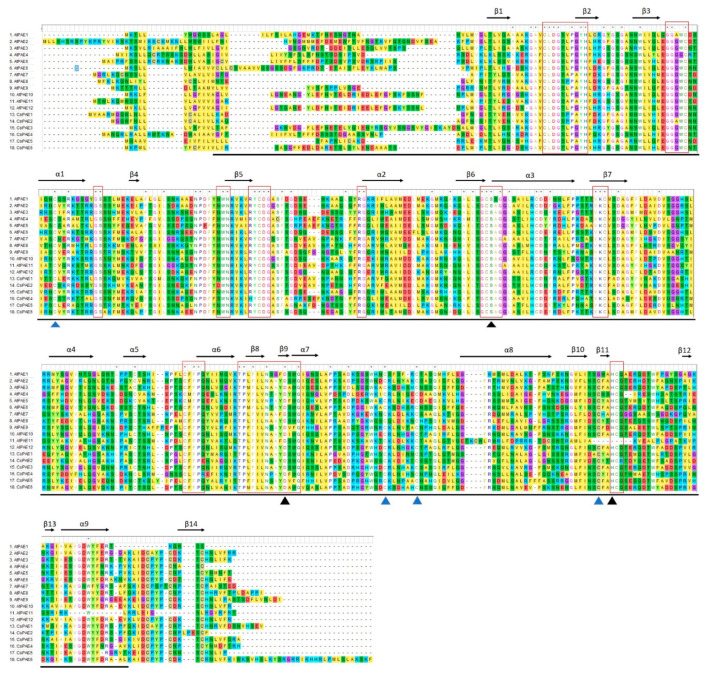
Alignment of the protein sequences of CsPAEs and AtPAEs. The Muscle tool in MEGA V7.0 was used for the alignment of full-length *A. thaliana* and *C. sinensis* PAEs. Red rectangles were used to highlight conserved PAE motifs. Secondary structural elements are shown by black arrows along the top of the protein sequences, with α and β corresponding to α-helices and β-strands, respectively. The S, D, and H active catalytic sites are marked using black triangles, while cysteine residues capable of disulfide bond formation are marked by blue triangles. Consensus sequences are marked an asterisks over the corresponding residues.

All six CsPAEs were predicted to contain an N-terminal signal peptide and a PAE domain (Pfam: PF03283) (Figure 2), and 9 α-helices and 14 β-strands in their secondary structures. Eleven conserved motifs were detected in the PAE domain of CsPAEs, including catalytic active site S, D, and H residues consistent with strong catalytic site conservation [11]. Indeed, conserved GCSxG, NxayDxxQ, and HCQ motifs were present within both CsPAEs and AtPAEs. Furthermore, these PAEs contain four cysteine residues that facilitate disulfide bond formation and enhance enzymatic thermostability (Figure 2).

### 2.4. Physical Distributions and Gene Structures of CsPAE Genes

**Figure 3:**
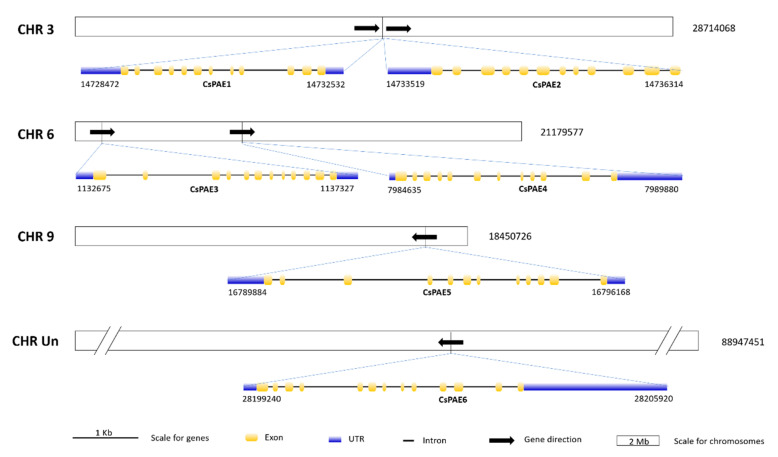
Chromosomal localizations and intron–exon structures of CsPAE genes. The localizations of CsPAEs were visualized by Mapchart V2.2, and the intron–exon structures of CsPAEs were determined using GSDS V2.0. Blue rectangles represent untranslated regions (UTRs) at 5 prime and 3 prime ends; yellow rectangles represent exons and blank lines represent introns; black arrows represent gene direction. The sizes of exons, introns, and UTRs are up to scale for a gene, while the sizes of chromosomes are scaled for chromosomes. The sizes of chromosomes are written on the right of them, and the gene localizations (the start and the end positions) are written below the genes.

The six *CsPAE* genes identified were located on three chromosomes (CHR3, 6, and 9) and the unassembled scaffolds. The exon–intron structures of *CsPAEs* were similar to those of AtPAEs with respect to the presence of many (10–12) introns [11]. *CsPAE1*–*5* were found to contain 11 introns, while *CsPAE6* contained 12 (Figure 3). Combining the chromosomal localization and the phylogeny (Figure 1), we conclude that *CsPAE1* and *CsPAE2* have suffered tandem duplication events in the evolution process, leading to the birth of a gene and neofunctionalization [36]. These two evolved into genes containing different CDSs (similarity: 82%), opposite acid/base preferences (basic vs. acidic), and significantly different intron sequences (Figure 3). The “newborn” *CsPAE2* could possess new functions in citrus.

### 2.5. CsPAE2 Was Inversely Induced by Xcc in CBC-Susceptible and CBC-Resistant Varieties

**Figure 4:**
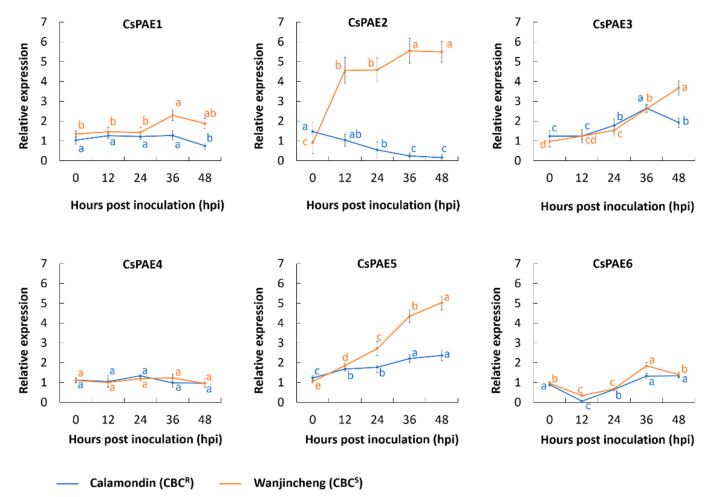
*CsPAE* expression profiles under the infection of *Xcc*. Wanjincheng (orange) and Calamondin (blue) were infected with *Xcc* for 0, 12, 24, 36, and 48 h, after which *CsPAE* expression was assessed via qRT-PCR, with *CsActin* being used for the normalization. Uninfected control samples were inoculated using LB medium. Data are means ± SEs. Duncan’s multiple range test was used to compare the data (*p* = 0.05), with three biological replicates per sample. The significance of the difference was marked by lowercase letters (a–e).

We next explored the functional roles of CsPAEs in response to biotic stress by assessing *CsPAE* expression patterns in leaves that had been infected by *Xcc* within 48 h post inoculation (hpi) by qRT-PCR. Specifically, we found that *CsPAE2*, *CsPAE3*, and *CsPAE5* were upregulated in the CBC-susceptible variety Wanjincheng. Of these genes, we found that *CsPAE2* was downregulated in the CBC-resistant variety Calamondin, whereas *CsPAE3* and *CsPAE5* were still upregulated in response to *Xcc* infection in Calamondin (Figure 4). This suggested that *CsPAE2* may be a potential susceptibility-related gene that plays a role in responding to *Xcc* infection. No significant changes in the expression of the other three *CsPAEs* were detected in response to *Xcc* infection in either Wanjincheng or Calamondin. Based on these results, we therefore selected *CsPAE2* as a potential candidate gene worthy of further study by reverse genetics strategies.

### 2.6. Overexpression of CsPAE2 Confers CBC Susceptibility

**Figure 5:**
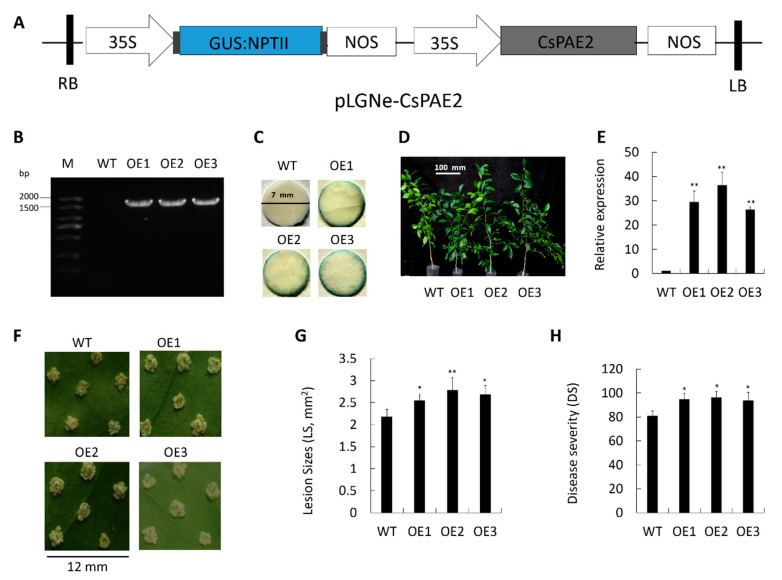
Assessment of *Xcc* responses in *CsPAE2* overexpression plants and WT plant. (**A**) Plasmid pLGNe-*CsPAE2* used for overexpression assay. NPTII, *NptII* gene; NOS, Nos terminator; GUS, glucuronidase; LB: left border; RB: right border. (**B**) PCR-mediated validation of transgenic plants. (**C**) GUS staining-mediated validation of transgenic plants. (**D**) Phenotypes of the transgenic plants. Scale bar = 100 mm. (**E**) The expression of *CsPAE2* in the indicated plants was measured via qRT-PCR, with *CsActin* for the normalization. At 10 dpi, disease symptoms on the leaves of WT and transgenic plants inoculated with *Xcc* were assessed (**F**); lesion size (LS) (**G**) and disease severity (DS) (**H**) were assessed. In (**F**), the scale bar = 12 mm. WT plants served as controls for statistical testing. Data are means ± SEs (*n* ≥ 3). Duncan’s multiple range test was used to compare data of OEs and WT, with three biological replicates per sample (* *p* < 0.05; ** *p* < 0.01).

In order to explore the role of CsPAE2 in the context of CBC, we next generated transgenic citrus plants overexpressing this protein using a *CsPAE2* overexpression plasmid that contained a glucuronidase (GUS) coding sequence under the control of a CaMV 35S promoter (Figure 5A). We confirmed the successful integration of *CsPAE2* in three overexpression plants (labeled OE1, OE2, and OE3) via both PCR and GUS assays (Figure 5B,C). These transgenic plants exhibited growth rates comparable to those of wild type plant (WT), but exhibited more bifurcation compared to WT plant (Figure 5D). When we assessed these three plants via qRT-PCR, we were able to confirm that they expressed significantly elevated *CsPAE2* levels (29-fold, 36-fold, and 26-fold of WT, respectively) (Figure 5E). The acupuncture method is used to assess and compare the resistance between OE plants and WT [2,37,38,39]. We found that these OE plants exhibited much larger lesions and more significant symptoms relative to WT (Figure 5F). Disease aggravation was most pronounced in OE2 plants, followed by OE1 and OE3, respectively. In OE2 plants, at 10 days post inoculation (dpi), lesions were approximately 127% the size of those in WT plants on average (Figure 5G). In addition, transgenic plants exhibited increases in disease severity (DS) by 16% (OE3) to 19% (OE2) relative to WT (Figure 5H). These results led us to conclude that CsPAE2 overexpression was sufficient to increase CBC susceptibility in transgenic citrus plants.

### 2.7. CsPAE2 Silencing Increases CBC Resistance

**Figure 6:**
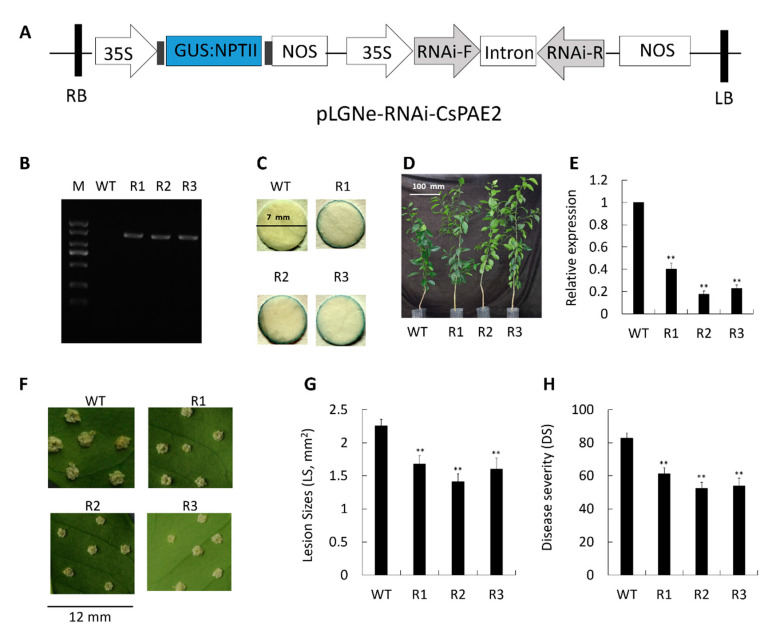
Assessment of *Xcc* responses in *CsPAE2* knockdown plants and WT plant. (**A**) Plasmid pLGNe-*CsPAE2*-RNAi used for RNAi assay. NPTII, *NptII* gene; NOS, Nos terminator; GUS, glucuronidase; LB: left border; RB: right border. (**B**) PCR-mediated validation of transgenic plants. (**C**) GUS staining-mediated validation of transgenic plants. (**D**) The phenotypes of transgenic plants. Scale bar = 100 mm. (**E**) The expression of *CsPAE2* in the indicated plants was measured via qRT-PCR, with *CsActin* used for the normalization. At 10 dpi, disease symptoms on the leaves of WT and transgenic plants inoculated with *Xcc* were assessed (**F**), and lesion size (LS) (**G**) and disease severity (DS) (**H**) were assessed. In (**F**), the scale bar = 12 mm. WT plants served as controls for statistical testing. Data are means ± SEs (*n* ≥ 3). Duncan’s multiple range test was used to compare data of OEs and WT, with three biological replicates per sample (** p < 0.01).

In order to expand upon the above results, we next knocked down *CsPAE2* via RNAi using appropriate constructs inserted into the pLGNe vector (Figure 6A). Three transgenic plants were obtained by PCR (R1, R2, and R3) (Figure 6B) and GUS staining (Figure 6C). Relative to WT, these transgenic plants exhibited higher growth rates (Figure 6D). Expression of *CsPAE2* in these plants was significantly reduced to 40%, 18%, and 22% of WT, respectively (Figure 6E). Upon infection with *Xcc*, the three mutants exhibited smaller pustules than those evident on WT plant (Figure 6F). We were therefore able to conclude that *CsPAE2* knockdown can significantly increase *Xcc* resistance in *C. sinensis*. Consistent with this, we observed significantly smaller lesion sizes (LS) in these three silenced plants (75%, 63%, and 71% of WT, respectively) (Figure 6G). Furthermore, an assessment of CBC severity indicated that these three transgenic plants exhibited markedly increased disease severity relative to WT plants (Figure 6H), with consequent decreases in DS of 26% (R1) and 35% (R3). These findings therefore confirmed that the knockdown of *CsPAE2* is sufficient to confer CBC resistance, thereby—together with the overexpression assay—indicating that *CsPAE2* is a CBC susceptibility gene.

## 3. Discussion

In the present study, we first employed a bioinformatics approach in order to comprehensively identify *PAEs* within the *C. sinensis* genome and to characterize their structures and gene expression profiles. We then further explored the functional relevance of these identified *CsPAEs* in response to *Xcc* infection, offering novel insights into the role of this gene family in the context of CBC resistance. This is the first study to our knowledge that has explored this topic.

*PAEs* compose a multi-gene family in higher plants, whereas in lower plants there is only one *PAE* gene copy [21]. This difference may be related to the differences in the acetylation modifications produced by these different enzymes. Lower plants may exhibit lower levels of acetylation, thereby necessitating reduced PAE enzymatic activity, whereas in higher plants pectin de-acetylation is a more complex process requiring lots of PAEs. We were able to identify in total six PAEs encoded in the *C. sinensis* genome, which is half the number detected in the Arabidopsis genome [11]. The difference in gene family sizes between the two species is related to the number of duplication events. In fact, five duplication events were detected in Arabidopsis, whereas only one was detected in *C. sinensis* [11]. We then used the sequences of these proteins to construct a phylogenetic tree, grouping these *CsPAEs* into three clades containing two *CsPAEs* per clade. Much like PAEs identified in Arabidopsis, we found that *CsPAEs* exhibited high numbers of introns (Figure 3). Intron-containing genes are known to increase their transcription more efficiently than non-intronic genes. These genes can also function as negative regulators of gene expression via generating intronic microRNAs capable of controlling PAE expression profiles in specific tissues or other regulatory contexts [40].

Several studies have shown that PAEs can regulate plant stress responses [11,12,19]. In Arabidopsis, the mutants of putative pectin acetyltransferase genes *PMR5* and *PMR6* are more susceptible to *B. cinerea*, whereas *PMR* mutants are less susceptible to powdery mildew infection [25]. *CsPAE* expression patterns may offer functional insights into their diverse roles in plants. As such, it is possible to better understand the role of *CsPAEs* in the context of plant defenses by quantifying changes in their expression in response to biotic stressors. In this study, we investigated CBC-responsive *CsPAE* genes via qRT-PCR, revealing *CsPAE2*, *CsPAE3*, and *CsPAE5* to all exhibit *Xcc*-dependent changes in their expression levels (Figure 4). Of these genes, we found that *CsPAE2* exhibited opposing expression patterns in Calamondin and Wanjincheng, being down-regulated in the former and upregulated in the latter upon *Xcc* inoculation (Figure 4). Using reverse genetic engineering strategies (overexpression and RNAi silence), we were then further able to determine that *CsPAE2* is a potential CBC susceptibility gene (Figure 5 and Figure 6). Regarding the phenotype changes, OE plants exhibited comparable growth rates, and RNAi plants exhibited faster growth rates compared to those of WT plants. Additionally, both the OE plants and RNAi plants possessed more bifurcation compared to WT plants (Figure 5D and Figure 6D). This result suggests that *CsPAE2* might also be involved in citrus growth regulation. 

This study highlighted the role of *CsPAE* genes in CBC development, thereby extending the current list of such CBC-related genes. However, many questions relating to this topic remain to be answered. For example, the mechanistic basis for *Xcc*-mediated induction of *CsPAE2* expression remains to be established, as does the functional role of *CsPAE2* during CBC infection. The observed differences in *CsPAE2* expression in Wanjincheng and Calamondin may provide some insights into the different cis-regulatory elements controlling its upregulation in these species [41]. Many future studies of how PAEs function in the context of plant immune responses are needed, and additional molecular and physiological research regarding the role of *CsPAE2* in CBC susceptibility are necessary in order to more fully understand the role of this gene involved in CBC development.

As such, these findings provide evolutionary insights into and functional investigations of the *CsPAE* gene family. This study also suggests that *CsPAE2* is a potential CBC susceptibility gene that negatively regulates CBC development and can be used to breed CBC-resistant citrus plants.

## 4. Materials and Methods

### 4.1. Annotation and Bioinformatics Analysis of CsPAEs

The *C. sinensis* genome and proteome were downloaded from CAP (http://citrus.hzau.edu.cn/orange) [34,35] and Phytozome (https://phytozome.jgi.doe.gov) [42,43]. A three-step semi-automated process was used to identify and annotate PAEs based on an initial query with 12 *A. thaliana* PAEs [36,44]. Functional and structural annotation were conducted using HMMER V3.3 (http://www.hmmer.org) [45,46], SMART (https://smart.embl.de) [47], and Gbrowse in CitGVD (http://citgvd.cric.cn) [48]. The identified *C. sinensis* PAEs were termed as CsPAE, and were numbered in the chromosomal order. Muscle was used for protein sequence alignment [49], and MEGA V7.0 was used for the maximum likelihood (ML) phylogenetic assay. The intron–exon structures and chromosomal localizations of *CsPAE* genes were visualized by Gene Structure Display Server V2.0 (GSDS, http://gsds.gao-lab.org) [50] and Mapchart V2.2 [51] respectively. Signal peptide and subcellular localization predictive analyses were done with SignalP V4.0 (http://www.cbs.dtu.dk/services/SignalP) [52] and CELLO V2.5 (http://cello.life.nctu.edu.tw) [53] respectively.

### 4.2. Plants and Bacteria

The National Citrus Germplasm Repository (Chongqing, China) was the source of the plants used in this study. *Xcc* assays were conducted using the Calamondin (*Citrus madurensis*) (CBC^R^) and Wanjincheng (*Citrus sinensis*) (CBC^S^) varieties, with the latter additionally being used for gene transformation. All plants were grown at 28 °C in a greenhouse. The *Xcc*YN1 strain was isolated from naturally infected sweet orange leaves, and was cultured at 28 °C using peptone-yeast extract-malt extract (PYM).

### 4.3. Xcc Assays

Xcc inducible expression patterns of CsPAEs were measured as in prior reports [39,54]. Briefly, the mature new leaves (approximately 3-month-old leaves) of Calamondin and Wanjincheng (roughly 10-year-old plants) were picked and placed in the culture plates, while keeping the petioles wrapped in cotton that was soaked in ddH_2_O. The leaves of these plants were then inoculated with 1000-fold dilution of *Xcc*YN1 (OD600 = 0.5, which is equivalent to 5 × 10^8^ CFU·mL^−1^) and then were incubated at 28 °C with a 16 h light/8 h dark photoperiod. Every 12 hpi, samples were collected for analysis through 48 hpi. Samples treated with LB medium was used as the control (CK). Primers are compiled in Table S3.

### 4.4. Plant Transformation

Overexpression plasmids were generated via initially amplifying the full-length *CsPAE2* coding sequences (CDS) using the following primers: F_OEC_ (CGGGATCCATGGGCCAATGGTTCAATCTTTTA), R_OEC_ (CGGAATTCTCAAAAGCAACTCTCTGGCAATGGGT). The PCR product was inserted into the vector pGLNe. Silencing vectors were constructed via amplifying a 300-bp fragment using the following primers: F-RIc (GCTCTAGAGGCGCGCCAATGAGCAGAAATTTAACCCA), R-RIc (CGGGATCCATTTAAATGCCAGCATCTGCAAAGCATTT). The amplified product was then inserted into the PUC-RANi so as to yield an RNAi sequence that was obtained and cloned into the pLGNe vector. Transformation of Wanjincheng shoot segments was conducted using *Agrobacterium tumefaciens* containing appropriate plasmids, as previously described [38,55].

### 4.5. Validation of Transgenic Plants 

The following primers were used to validate the overexpression of transgenic plants: F_OED_ (CGACACGCTTGTCTACTCCA) (targeting the 35S promoter) and R_OED_ (CGGAATTCTCAAAAGCAACTCTCTGGCAATGGGT) (targeting CDS of C terminal). The following primers were used to validate RNAi transgenic plants: F_RD_ (TGCAGATGCTGGCATTTAAATGTGTAA) (targeting RNAi-F fragment) and R_RD_ (CTACGACCGGGATCCAAATACCTGCAAA) (targeting the left border of pLGNe). A 1705-bp and a 1454-bp fragment can be amplified from the OE and RNAi plants respectively; no amplification from WT. A histochemical approach was used to measure GUS activity in these transgenic plants [37,55]. The expression of *CsPAE2* was then measured in transgenic plants using the F_RT_ and R_RT_ primers (Table S3), with WT serving as controls in all of these assays. Finally, 3 *CsPAE2* overexpression plants and 3 *CsPAE2* RNAi plants were obtained.

### 4.6. Measurement of CBC Resistance

The resistance of transgenic plants to *Xcc* infection was assessed with acupuncture inoculation method as protocol in previous reports [2,38,55]. Briefly, 6 healthy mature leaves from each plan were obtained, and a pin (0.5 mm in diameter) was used to generate 6 punctures in the surface of each leaf. A total of 1 μL of an *Xcc*YN1 bacterial suspension (5 × 10^5^ CFU·mL^−1^) was then used to inoculate each of these pinprick spots. At 10 dpi, leaves were imaged, and ImageJ (NIH, Bethesda, MD, USA) was used to analyze the DS and LS. The DS was calculated as in previous studies [55].

### 4.7. qRT-PCR

The frozen tissue samples were ground, and the total RNA was isolated using a miniprep kit (AidLab, Beijing, China) and then reversely transcribed to cDNA (TaKaRa, Dalin, China). Thermocycler settings of qRT-PCR were: 4 min at 95 °C; 40 cycles of 95 °C for 10 s, 56 °C for 30 s, and 72 °C for 30 s. Relative expression was measured via the 2^−∆∆CT^ method [56]. *CsActin* (CAP ID: Cs1g05000, GenBank: GU911361.1) was used to normalize relative expression. Assays included three biological and three technical replicates.

### 4.8. Statistical Analysis

SPSS V22 (IBM, Chicago, IL, USA) was used for all statistical testing. The differences were evaluated using variance (ANOVA) based on Duncan’s multiple range test was used to analyze the significance of differences. In the test, *p* < 0.05 and *p* < 0.01 were the thresholds of significance and extremely significance respectively.

## Figures and Tables

**Table 1:** List and details of PAEs identified in *C. sinensis* genome.

Name	CAP ID	AA NO.	MW (DD)	PI	EST NO.	Annotation
CsPAE1	Cs3g10410.1	399	43,822.24	8.68	12	CAP, P, EST
CsPAE2	Cs3g10420.1	386	42,542.05	5.86	1	CAP, P, EST
CsPAE3	Cs6g01740.1	423	47,321.84	9.01	3	CAP, EST
CsPAE4	Cs6g06280.1	424	47,569.82	6.41	1	CAP, P, EST
CsPAE5	Cs9g17480.1	397	44,834.28	8.39	2	CAP, P, EST
CsPAE6	orange1.1t01789.1	441	49,247.08	8.24	2	CAP, P, EST

All PAEs are listed. MW: molecular weight. AA: amino acid. PI: isoelectric point. In annotation: P: Prediction by phytozome; CAP: citrus annotation project (CAP) prediction. EST: genes with EST hits.

## Supplementary Materials

Supplementary materials can be found at https://www.mdpi.com/1422-0067/21/24/9429/s1. Table S1. ESTs of *CsPAEs*. The best EST hits were extracted based on tBlastn from an EST dataset downloaded from NCBI. Table S2. The gene, CDS, and protein sequences of CsPAEs. Table S3. qRT-PCR primers used in this study. Primers were designed with the NCBI primer blast tool, with a *C. sinensis* mRNA database being used to check specificity.
